# Ciliate Morpho-Taxonomy and Practical Considerations before Deploying Metabarcoding to Ciliate Community Diversity Surveys in Urban Receiving Waters

**DOI:** 10.3390/microorganisms10122512

**Published:** 2022-12-19

**Authors:** Yan Zhao, Gaytha A. Langlois

**Affiliations:** 1 College of Life Sciences, Capital Normal University, Beijing 100048, China; 2 Department of Science & Technology, Bryant University, Smithfield, RI 02917, USA

**Keywords:** ciliated protozoa, urban receiving waters, DNA metabarcoding, molecular-based taxonomy, water quality, biomonitoring

## Abstract

Disentangling biodiversity and community assembly effects on ecosystem function has always been an important topic in ecological research. The development and application of a DNA metabarcoding method has fundamentally changed the way we describe prokaryotic communities and estimate biodiversity. Compared to prokaryotes (bacteria and archaea), the eukaryotic microbes (unicellular eukaryotes) also fulfill extremely important ecological functions in different ecosystems regarding their intermediate trophic positions. For instance, ciliated microbes (accounting for a substantial portion of the diversity of unicellular eukaryotes) perform pivotal roles in microbial loops and are essential components in different ecosystems, especially in water purification processes. Therefore, the community composition of ciliated species has been widely utilized as a proxy for water quality and biological assessment in urban river ecosystems and WWTPs (wastewater treatment plants). Unfortunately, investigating the dynamic changes and compositions in ciliate communities relies heavily on existing morpho-taxonomical descriptions, which is limited by traditional microscopic approaches. To deal with this dilemma, we discuss the DNA-based taxonomy of ciliates, the relative merits and challenges of deploying its application using DNA metabarcoding for surveys of ciliate community diversity in urban waterbodies, and provide suggestions for minimizing relevant sources of biases in its implementation. We expect that DNA metabarcoding could untangle relationships between community assembly and environmental changes affecting ciliate communities. These analyses and discussions could offer a replicable method in support of the application of evaluating communities of ciliated protozoa as indicators of urban freshwater ecosystems.

## 1. Introduction

Natural river systems in good condition often have the capability of biological self-purification, which complements the physical-chemical processes and plays a key role in maintaining river health. This capability is highly dependent on the action of complex microbial communities that consist of decomposers (bacteria and fungi, utilizing the dissolved organic matter) and consumers (bacterivorous and carnivorous protists, such as ciliates, flagellates, and amoebae feeding on dispersed bacteria and other organisms) [1,2]. In urban rivers with significant nutrient pollution [3], the capacity of river self-purification might be dysfunctional and cause a higher potential health risk in the city. Considering that a river’s health is related to water supply and sanitation and is of utmost importance [4], the assessment of a river’s condition is paramount. 

As one of the most diverse protist groups, previous studies reveal that ciliates play an important ecological function in the self-purification and material cycling processes of urban water bodies [5,6,7,8]. Understanding the strong correlations of different ciliated bioindicators with the respective self-purification stages could further enhance and serve as a basis for developing and utilizing ciliates as bioindicators during bioremediation and water purification. However, little attention has been given to untangling these processes by specifying the assemblage patterns of specific taxa in response to different pollution levels, which severely limits the understanding of the value and variety of the ecological functions of the ciliates and their potential bioindicator applications. One explanation is that the traditional ecological and biomonitoring ciliate studies have usually involved visual surveys and capture, followed by morphological identification, which relies heavily on professional taxonomists [9,10]. Recent advances in next-generation sequencing (NGS) technology have triggered and facilitated intense research on microbial ecology, which has revealed a ‘hidden world’ of microbial eukaryotic diversity in the oceans [11,12]. Thus, we expect that DNA metabarcoding also has an enormous potential to facilitate the study of ciliate biodiversity and an understanding of their community assembly in urban freshwater ecosystems. Hopefully, the use of this method would make it easier to evaluate urban water quality and provide ongoing biomonitoring strategies using ciliated species. 

Although the advent of metabarcoding holds promise for such analyses, researchers should be aware that the limitations and biases inherent in sequencing-based analyses and proceed to untangle the signal of taxonomic diversity only after the distortion factors of this method have been minimized as much as possible. Therefore, this review aims to discuss the relative merits and challenges of deploying DNA metabarcoding for surveys of ciliate diversity and their population dynamics in urban water bodies. Only then would the metabarcoding method contribute significantly to our understanding of the community assembly of ciliates, confirm their potential roles in ecological function and biogeochemical cycles in urban ecosystems, and result in the stimulation of a broader interest in ecology of microbial eukaryotes [13,14]. 

## 2. Ciliates Involved in Self-Purification in Treatment Plants and Urban Waterbodies

Self-purification is a natural process that involves the recycling of pollutant materials (released though anthropogenic activities) by various complex processes related to physical, chemical, and biological mechanisms [15]. The biological process of self-purification includes both prokaryotic (bacterial degradation) and eukaryotic microbes (unicellular protists). The eukaryotic microbes are well represented by ciliate lineages due to their strong grazing impact on the bacteria and microalgae. Therefore, ciliates could be used as bioindicators in a wide range of contexts, from monitoring ecosystem restoration to addressing environmental pollution. Many enthusiastic ciliatologists, such as Colin R. Curds and Wilhelm Foissner, have contributed significantly to these findings [5,6,8,16,17].

In China, Shen Yunfen, a skilled taxonomist of protozoa, has contributed extensively to the taxonomic information of protozoa in the biomonitoring of urban waterbodies during the 1980s and 1990s. As documented in “Modern BiomonitoringTechniques Using Freshwater Microbiota”, Shen and her colleagues provide a superb guide to the bioassessment of protozoan communities [18]. However, the reference has not been widely used in this field, because the number of experts (both ecologists and taxonomists) is insufficient. Although 749 protozoa taxa are recorded in detail, with their descriptions, numbers and keywords strongly correlated to chemical pollution index.

Recently, the potential for the performance monitoring of ciliate communities in wastewater treatment plants (WWTPs), using a molecular approach, has been widely demonstrated [19,20,21]. Although the molecular approach, including the metabarcoding of ciliate communities, may not be a faster and less costly monitoring tool for water quality and chemical pollution, the premise is that the method could accurately identify and quantify ciliate communities at the species level. At this time, however, the focus on ciliate communities is still hampered by identification difficulties and an inadequate capability of quantifying species abundance.

Unfortunately, different species show different lifestyles and feeding strategies, and have different roles of agency for environmental monitoring and other ecological functions. Thus, the lumping together of similar species at the genus or higher taxonomic levels as an indicator category, while lacking knowledge of their autecology, could cause unreliable predictions. We must avoid the dilemma encountered by bacteria studies in which their identification now relies heavily on sequence-based taxonomy that is not well correlated with traditional taxonomic approaches, which results in problematic and confusing interpretation [22]. Warren, et al [23] set forth recommendations for best practices regarding the documentation of ciliate biodiversity, which was aimed at bridging gaps between sequence-based taxonomic methods and more traditional approaches.

With the advent of the molecular era, we need to make ciliate metabarcoding become a routine tool, rather than an optional rough tool in our biodiversity survey of urban waterbodies. Therefore, first and foremost, the reference data linking valid species identification and the corresponding molecular data should be built. That is, researchers of ciliates in urban waterbodies should replicate the work of Shen’s team [18] in the support of the classical and traditional taxonomic identification; meanwhile, a reliable molecular reference database should be provided.

## 3. Traditional Ciliate Taxonomy and Its Investigation in Freshwater Systems

Ciliated protozoa that inhabit an invisible world were first discovered and described by van Leeuwenhoek in 1677 [24,25]. However, the discovery of these protistans did not attract much scientific interest over the following centuries because their taxonomic descriptions failed to represent the taxonomy paradigm forged by Carl Linnaeus. The foundations of the Linnaean taxonomy are morphological characters used to diagnose genera and species. Therefore, the Linnaean category of ciliates relied heavily on high-resolution microscopy. After a long delay, taxonomical studies of ciliates were triggered by the invention and progress of better microscopes [26]. So far, more than 4500 free-living ciliates have been described in detail, based on morphological traits, with reliable identifications and modern nomenclature, including those that inhabit fresh water (lakes, reservoirs, rivers, and ponds), brackish water, and salt water [9,16,27,28]. Subsequently, the science of ciliate taxonomy provides a foundation for comparative studies from molecular biology to ecology, and these disciplines are further encouraged and have become standard practice in taxonomy [29,30,31,32,33].

Aligned with the “microbial loop” concept, many researchers have been stimulated to study the ecological aspects of ciliated protozoa, both in marine and freshwater environments [12,34,35,36,37]. However, often, reliable identification was missing due to insufficient attention to species identification, and named species referenced only to line drawings. Moreover, most of the species were not described or sufficiently characterized by professional taxonomists, and there were insufficient dependable guides available that included detailed descriptions or excellent figures [16]. 

The situation improved during the nineteenth century, when numerous professional taxonomists devoted themselves to ciliate identification in freshwater systems [16]. The common, dominant, and rare species of heterotrichs, scuticociliates, hymenostomes, oligotrichs, choreotrichs, and hypotrichs were identified and characterized in detail, and easily confused species identifications were revised or recommended for later revisions. 

However, an illustrated guide to the ciliate species in freshwater still could not provide high efficiency of species identification to microbial ecologists, who preferred to avoid intense and time-consuming taxonomic work. In recent years, the development of an integrative taxonomy of ciliates has provided a universal framework of DNA taxonomy, and non-taxonomists, especially ecologists, would likely utilize DNA analyses for routine species identification [10,38,39]. The genetic species identification method could also greatly reduce the subjective identification of ciliates by both taxonomists and non-taxonomists. However, this approach requires the existence of curated nucleic acid sequence databases. Therefore, building and maintaining a stable and vital taxonomic framework still seems a long way off, and it is still necessary to rely on the traditional approach of morphological taxonomy and professional taxonomists. 

## 4. Phylogenetic Taxonomy and DNA Barcoding of Ciliates

Dobzhansky famous’s dictum that, “Nothing in biology makes sense except in the light of evolution” reflected the perspectives of Darwinian theories of evolution [40]. These theories have had profound and immediate impacts on different biological disciplines, and taxonomy is no exception. Phylogenetic taxonomy has constructed taxonomic definitions based on phylogeny, and this was a revolution of the modern Linnaean taxonomy that was initiated by Hennig [41], with the concerns of common descent and specific evolutionary interpretations in taxonomy [42]. From the perspective of phylogenetic taxonomy, the taxa are unified by their common evolutionary descent, while the Linnaean taxa shared the morphological and ontogenetic characteristics. Phylogenetic taxonomy is devoted to the identification of taxa boundaries and attempts to deal with evolutionary relationships among deeper taxa in the framework of the evolutionary (phylogeny) tree, which can be further divided into morphological phylogenetics and molecular (genetic) phylogenetics.

Morphological phylogenetics are dedicated to finding homological phenotypic (typically morphological) traits that derive from the common descent, thus enhancing our knowledge of both living and extinct biodiversity and their evolutionary history in the taxonomical study of the genus and species. However, with the advent of the molecular era from the 1960s onwards, scientists sought to implement the usefulness of molecular and genetic data for phylogenetic inference and have taken phylogenetic studies in a new direction, that of molecular phylogenetics [43,44]. Studies in molecular phylogenetics provide deeper phylogenetic insights into the mitochondrial gene cytochrome *c* oxidase I (*cox 1*) in resolving species-level assignments, which was designed as DNA barcoding to accelerate taxa identification. The taxonomic implication of DNA barcoding is performed under the premise that genetic variation of a standardized gene region between species exceeds the variation within species [45,46]. 

Given the inherent properties of ciliates of single celled, diverse, and complex morphology, their traditional taxonomy is complex and difficult [47]. Thus, phylogenetic taxonomy and DNA barcoding at low taxonomic levels of ciliates could offer a promising system for ciliatology. Subsequently, the Protist Working Group (ProWG) initiated the CBOL (Consortium for the Barcode of Life) project and called for a reference library based on standard protistan barcodes [48]. Pilot studies indicated that some genomic regions (*cox 1*, *cob*, SSU-V4/V9, and LSU-D1-D2 regions) with high and moderate evolutionary rates provide a bio-barcode for identification and could be selected as species-specific barcodes [38,39,49,50,51,52,53,54]. Taken together, these results support the concept that the *cox 1* marker shows significantly better performance than other candidates for the accurate identification of closely related species. However, the high variability of the *cox 1* marker made it impossible to design universal primers suitable for all ciliate lineages, resulting in only a small number of reference data available (Zhao, et al. 2018). In contrast, there are numerous reference sequences of SSU, and the conserved sequences flanking the hypervariable V4 and V9 regions facilitates the design of universal primers [39,55,56]. Consequently, high-throughput sequencing of the SSU-V4/V9 sequences are extensively used to survey eukaryotic microbial communities, including those of the ciliate species [12,21,54,57]. 

Although the research studies reveal the ecological importance of ciliates in various environments, the ecology of individual species is unknown. Therefore, building ecological models of ciliate diversity and their relationships between taxonomic and functional components remains an exceptionally great challenge [58,59,60,61,62]. However, these SSU-based DNA metabarcoding studies ignore the fact that the inconsistencies between SSU-based and morphology identifications are likely due to the remarkably diverse and comparatively complex morphology of ciliates [47,63]. The rate of SSU molecular and morphological evolution in ciliates did not match in the process of speciation, and sometimes, the morphologically indistinguishable taxa might fall under the category of cryptic speciation, which in turn causes the confusion of taxonomic concepts in ciliatology [39]. 

Undoubtedly, homology is clearly at the center of evolutionary interpretations in ciliate taxonomy. However, it is still a challenge for amateur taxonomists to measure homology in the morphology and molecular characters. Therefore, both the phylogenetic taxonomy and the DNA barcoding of ciliates intensify the need for reliable DNA reference libraries that build on a solid taxonomic foundation.

## 5. High Throughput Sequencing and Metabarcoding

The advent of NGS as represented by Illumina’s sequencing platforms has revolutionized the study of microbial biodiversity [64,65]. The release of Ilumina’s MiSeq and HiSeq platforms (the former provides substantially longer reads than the latter) provide cost-effective tools for analyses focused on targeted gene sequencing using reversible-terminator sequencing-by-synthesis technology, and the two platforms have become the most prevailing sequencers for microbial community analysis [66,67]. Here, DNA metabarcoding extends DNA-based species identification and is realized by the high-throughput sequencing of the targeted gene that was used for DNA barcoding. That is, DNA metabarcoding is derived from DNA barcoding, using the same principles under the impetus of NGS technology, which could produce substantial amounts of data with high efficiency and throughput [68]. 

However, there is a need to recognize that NGS technology is a double-edged sword, considering that the limits and sequencing errors of NGS data from different platforms are almost inevitable. Therefore, there is the dual risk of increased difficulty in identifying a confident genetic variation from base calls, coupled with the use of genetic variation in barcoding studies regarding species assignment with an incomplete reference databases. The fast development of the NGS technology and the demand for DNA-based species identification challenges the design and the deployment of metabarcoding studies. The research approach requires not only identifying systematic sequencing errors and distinguishing these errors from true genetic variation using suitable bioinformatics software, but must also build an accessible barcode reference library with a reliable taxonomic assignment created by highly specialized taxonomists.

## 6. Factors That Might Sistort Ciliate Biodiversity Assessments Based on Metabarcoding

Biodiversity studies must identify the species to further understand different ecological processes and functions that depend on that species. Therefore, species identification and species discovery are a necessary step essential for ecological studies. DNA metabarcoding can be used as a complementary approach for taxonomic resolution, based on genetic principles in biodiversity assessment. 

**Figure 1:**
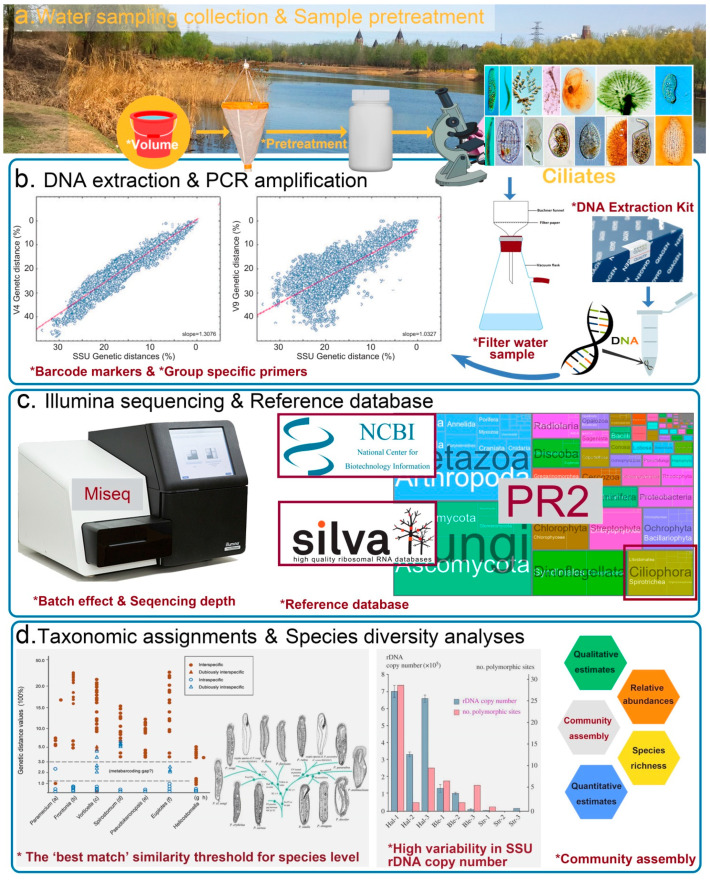
The flow chart of the metabarcoding processing steps (water sampling collection, sample pretreatment, DNA extraction, and PCR amplification, NGS-Illumina sequencing for example, reference database selection, and taxonomic assignment) of ciliate biodiversity. Factors that might distort biodiversity assessments are marked with an asterisk. The interior pictures in terms of “Barcode markers & Group specific primers”, “The ‘best match’ similarity threshold for species level” and “High variability in SSU rDNA copy number” are modified after the work of Dunthorn et al. [55] and Zhan et al. [70] and Wang et al. [71].

However, the metabarcoding of ciliate diversity is still in its infancy and is prone to several limitations when conducting empirical studies [69]. In urban areas, the DNA metabarcoding of ciliate communities in waterbodies can be summarized in the following experimental steps: (1) field sampling and pretreatment, (2) DNA extraction, (3) PCR amplification of selected DNA barcoding markers, (4) sequencing amplicons on NGS platforms, and (5) bioinformatic analyses (Figure 1). Each step can potentially introduce its own sources of artifacts and yield a biased estimate of the true patterns in biodiversity. Therefore, the most critical issue facing the metabarcoding studies of ciliates is to standardize each step to produce high-quality and reproducible results in order for it to become a routine tool for biodiversity survey and ecological studies. By identifying the pitfalls and challenges for each step in the process, some suggestions can be made for moving toward standardization.

### 6.1. Field Sampling and Pretreatment

A sufficient sampling effort is important to account for representative microbial biodiversity in metabarcoding studies, and researchers should make sure the sampling design is appropriate and standardized to accurately detect and identify a broad range of target species [72]. For ciliate metabarcoding studies, the field work in urban rivers includes capturing water samples with predetermined volumes, concentrating them with different processing steps (precipitation, centrifugation, or filtration), and avoiding contamination (Figure 1a). 

Sample volumes for ciliate metabarcoding studies range widely from 500 ml to 30 L [73,74,75,76,77,78,79]. However, recent metabarcoding studies of fish and amphibians demonstrated that the representation of their biodiversity is strongly influenced by the volume of water filtered [80,81]. Thus, a greater predetermined volume is needed to detect ciliate diversity. On the other hand, the larger volume would increase the filter burden. Our suggestion is to collect 30 L of water from surface waters, with triplicate sample replication using a bucket, and to pre-filter them with a ~150 µm filter screen to reduce the subsequent filtration time and to increase the throughput volume of the target species (by removing macrozooplankton). All enriched 1 L samples should be frozen or dried within ~12 h and transported to the laboratory to avoid shifts in the community dynamics of ciliates (with a shorter interval of time in summer, ~8 h). 

Some studies suggest that filter materials and pore size might also significantly impact DNA retention and therefore, the selection of appropriate filters and filter strategies is essential [82]. Here, we recommend that the entire sample should be filtered through sterile filter membranes of 47 mm diameter and 0.45-μm pore size (filter materials: cellulose-nitrate filtration), in combination with a vacuum pump. 

It is important to emphasize that care should be taken between sampling rounds, and during sampling, pre-filtering, and the subsequent filtering processes. Sampling equipment should be cleaned carefully before each new round to avoid contamination.

### 6.2. DNA Extraction

There are many commercial kits, as well as noncommercial methods and modified extraction protocols for DNA extraction (Figure 1b). The quality and quantity of the extracted DNA have been evaluated sufficiently in earlier research [82,83]. As the DNeasy Blood & Tissue kit yielded a higher concentration and quality of the extracted DNA, we recommend this commercial kit for the successful implementation of ciliate metabarcoding surveys.

### 6.3. PCR Amplification and the Choice of Barcode

The polymerase chain reaction (PCR) could amplify a substantial quantity of DNA fragments for different NGS platforms. There are some key options and considerations to consider in the PCR amplification step of metabarcoding analyses, particularly the fidelity of polymerase and the selection of barcode markers.

Detecting intraspecific and interspecific sequence variation is critical to the metabarcoding studies of ciliates. However, during enzymatic DNA amplification, the choice of DNA polymerase could significantly affect error rates of the nucleotide sequence [71,84,85]. The most used polymerase, the exTaq polymerase (Takara), also shows potential amplification artifacts, which could confound further biodiversity estimates in metabarcoding experiments [71]. Considering the cost, fidelity, and PCR efficiency factors, we recommend using the HotStart Taq DNA Polymerase to minimize PCR-based amplification artifacts in environmental metabarcoding analysis. There are other factors, such as PCR cycling conditions, that should be optimized for particular samples.

Careful selection of barcode markers is one of the most crucial steps, which can ensure the realization of the simultaneous identification of different taxa in environmental metabarcoding analyses. However, the standard *COI* barcode marker for taxonomic identification is not adaptable on the Illumina platform (and other NGS platforms) due to its resolution requirement of sequencing contigs longer than 500 bp [86]. Besides, there are no sufficient reference data for morphologically described species and universal primers that have been used for *COI* gene in ciliates [39]. Fortunately, various studies have shown that the universally present V4 and V9 regions of 18S rDNA barcodes are alternatives to the 500-nucleotide amplicon in ciliate high-throughput-sequencing metabarcoding studies guided by curated SSU rDNA databases (PR2 and SILVA database) [54,60,61,62,74,87,88,89]. Further studies demonstrate that the V4 SSU rRNA fragment is taxonomically informative and could generate comparable results to those of full-length 18S rRNA gene sequences; it also carries more phylogenetic signals in the phylogenetic relationships and the morphological evolution of ciliates than does the V9 region [39,55] (Figure 1b). Thus, we recommend that the V4 SSU rRNA fragment be used as a DNA barcode in metabarcoding analyses to investigate the diversity and taxonomic assignment of ciliates in field surveys. The tagging strategy is also highly recommended to reduce the cost due to its ability to allow hundreds of samples to be pyrosequenced in multiplex [90]. Group-specific primers that accurately reflect the water quality are also recommended to be designed for monitoring species.

### 6.4. Amplicons Sequencing on NGS Platform

DNA metabarcoding studies are susceptible to batch effects, which are critical and could bring common variations and artifacts in high-throughput experiments [91]. Thus, we encourage researchers to multiplex several independent samples (8–10) in one run (each PCR product to be tagged by the designed unique eight-base barcode to the V4 primers and prepared for the multiplexing of different samples) and sequence all samples in the same sequencing batch using paired-end Illumina MiSeq sequencing (which processes longer contigs than HiSeq, Figure 1c). The sequence depth should be reasonable and sufficient, based on the empirical data.

## 7. Bioinformatics 

The general steps for bioinformatic processing of the NGS platforms, such as MiSeq sequencing data, include data filtering, sequence classification, and the downstream analyses of multivariate statistical procedures. However, there is no single universal and streamlined workflow that can be satisfactorily used to treat DNA metabarcoding data from raw sequences into taxonomic assignment and biodiversity analyses. Each step faces technical complexities, and the result might be impacted by different parameter selections [86,92].

### 7.1. Data Filtering

Specifically, the process of the data filtering includes sample demultiplexing, error correction, merging read pairs, quality filtering, chimera filtering, sequence dereplication and singleton removal to finally obtain unique sequences (ASVs, amplicon sequence variants) of each sample. There are many pipelines that have been developed for dealing with the above processes, including DOTUR [93], MOTHUR [94], QIIME [95], USEACH [96], and VSEARCH [97]. There is no need to determine which methods outperform the others because the core module of these software options rely heavily on different algorithms to disentangle biological variation from amplicon sequencing errors. There are two popular algorithms, named UPARSE [98] and DADA2 [99]. Here, we recommend open-source software DADA2 for correcting amplicon errors without constructing OTUs (operational taxonomic units), but instead using ASVs (amplicon sequence variants) delimitation. One reason for not lumping together all similar sequences based on their OTU clustering threshold (97% similarity) would be the change of misinterpreting biological variation; another is that there is no universal ribosomal sequence variant (the threshold value) for all ciliates of different genera that could guarantee ecological coherence or evenness [70] (Figure 1d). Thus, we support the shift from the OTUs method to the ASVs method in ciliate metabarcoding studies to avoid the influences of artificial taxonomic cutoff [100].

### 7.2. Sequence Classification

The next key process is the taxonomic assignments of ASVs. Creating a high-quality taxonomic reference library has consequently become the most important limitation of a metabarcoding study, since metabarcoding values are critically dependent on the reference database to assign ASV sequences to identified taxa [62,68]. There are some existing reference databases, such as NCBI, SILVA, and PR2 to choose from; however, some challenges remain [88,99] (Figure 1c). The above databases sometimes fail to give congruence annotations of the ASVs and result in consistent taxonomic compositions of the same metabarcoding survey due to their incongruent taxonomic resolution at different taxonomic levels [61,62]. Subsequently, the manually curated EukRef-Ciliophora database (assimilated into PR2 database) by experts (not ciliate taxonomists) becomes the current best possible choice of our ciliate metabarcoding survey [61,62]. Other lineage-specific datasets, such as EukRef-excavates, are based on that approach for improved identification [101]. Therefore, we encourage researchers to assign ciliate ASVs and sequences of other eukaryotic microbes using a EukRef-classifier following EukRef guidelines professionally (available at https://github.com/eukref/curation, accessed on 25 October 2022; Figure 1c).

Some ciliate groups are known to be prone to misclassification. An extensive genetic diversity of ciliates in different ecosystem has been unveiled [71,102] (Figure 1d). The reason for this tendency is twofold: a large proportion of unknown or cryptic ciliates show high genetic divergences of the SSU rDNA among different ciliate linages, plus conflicts between morphology and molecular data often confuse phylogeneticists, as well as the taxonomists, even if they are the same person [61,63,70,103]. Therefore, the EukRef-Ciliophora curators should make sure that the reference sequences are obtained from taxonomical sequencing verified by ciliate taxonomists. 

Furthermore, the conspicuous disequilibrium of the proportion of rare and abundant species in the existing references is also common, thereby potentially rendering it difficult to analyze and interpret the data of rare species. Here, the guidelines suggest that researchers who have received professional training should build local reference databases for special habitat needs, instead of only selecting the existing ones. For example, we could build a local SSU reference library for ciliate metabarcoding studies in urban waterbodies. If the targeted ciliates described by Professor Shen Yunfen and her colleagues [18] are selected, their sequences could only be stored if the sequences were obtained from taxonomically verified data, and the library allows for continuously adding data of new taxa, as well as periodically overhauling the taxonomic classifications.

Therefore, the DNA-based identification can only work if all ASVs are correctly assigned to species, and the appropriate reference libraries must be chosen wisely in the process of taxonomic assignment. Of equal importance, researchers should not assign species using Blast searches by choosing an arbitrary cut-off value for ciliate species of different genera.

## 8. Conclusion: Developing Universal and Standardized DNA-Based Methods for Ciliate Studies in Urban Waterbodies

Taxonomy is essential for the fundamental understanding of species biodiversity and their ecological function, even though most of the biodiversity studies avoid the burdens of taxonomy due to their being costly, time-consuming, and relying on specialized taxonomists. However, the current taxonomy crisis could further lead to a biodiversity crisis. To solve the dilemma, contemporary taxonomists have a duty to develop universal and standardized DNA-based methods as parameterization suitable for the integrative taxonomy, which would provide effective, standardized, and robust species identification analyses for ecology and other related disciplines [32,104,105]. Ciliate taxonomists are no exception.

Ciliates are important trophic links in aquatic environments, and their ecological functions have received increasing attention [106]. However, the reality of the scenario is that taxonomical difficulties give rise to a serious lag in ciliate ecology. Despite extensive efforts having been devoted to describing and revising ciliate identification and providing excellently illustrated guides [16,107], the microbial ecologists are still frustrated by traditional microscopic approaches, which focus on morphological features. Therefore, ciliate taxonomists should pay more attention to easy and efficient methods developed for delimiting species to help ecologists out of the current taxonomy crisis.

With the emergence of the digital era, developing universal and standardized DNA-based methods for ciliate ecologists or other related researchers seems attainable. The project mainly comprises the following steps: (1) delimiting ciliate species based on many disciplines as a standard practice for integrating a variety of data, including morphological, molecular (18S must be included), and ecological characteristics; (2) discussing related or cryptic species broadly, if their morphological features are limited, and classifying the species carefully; and (3) building comprehensive 18S reference libraries, as they are the first and most important step toward metabarcoding studies. Simultaneously, researchers must be careful to use appropriate statistical approaches for estimating species richness (qualitative estimates) and relative abundance (quantitative estimates) of ciliate species during community assembly (Figure 1d).

Our premise is that refinement and standardization of metabarcoding protocols show considerable promise for ciliate diversity surveys as a means of monitoring water quality in urban rivers. Besides, it is only after a specific ciliate data reference exists that metabarcoding approaches could promise to be a complementary alternative that will enable microbial ecologists to gain more comprehensive insights into changes in ciliate biodiversity in a brief time. Furthermore, the expanded data acquisition and methods for synthetic analyses would be valuable and meaningful for using ciliates for the assessment of environmental impacts, water quality monitoring, and biosensors for heavy metal or other pollutants. Thus, there is work yet to be done to make metabarcoding more effective, accurate, repeatable, and informative to relate the species structure of ciliated protozoan communities and unravel their significance to water quality in urban rivers.
